# Incidence of muscle wasting in the critically ill: a prospective observational cohort study

**DOI:** 10.1038/s41598-023-28071-8

**Published:** 2023-01-13

**Authors:** Ondrej Hrdy, Kamil Vrbica, Marek Kovar, Tomas Korbicka, Radka Stepanova, Roman Gal

**Affiliations:** 1 Department of Anaesthesiology and Intensive Care Medicine, University Hospital, Jihlavska 20, 625 00 Brno, Czech Republic; 2 Department of Anaesthesiology and Intensive Care Medicine, Faculty of Medicine, Masaryk University, Brno, Czech Republic; 3 Department of Pharmacology, Faculty of Medicine, Masaryk University, Brno, Czech Republic

**Keywords:** Medical research, Risk factors

## Abstract

Loss of muscle mass occurs rapidly during critical illness and negatively affects quality of life. The incidence of clinically significant muscle wasting in critically ill patients is unclear. This study aimed to assess the incidence of and identify predictors for clinically significant loss of muscle mass in this patient population. This was a single-center observational study. We used ultrasound to determine the rectus femoris cross-sectional area (RFcsa) on the first and seventh day of ICU stay. The primary outcome was the incidence of significant muscle wasting. We used a logistic regression model to determine significant predictors for muscle wasting. Ultrasound measurements were completed in 104 patients. Sixty-two of these patients (59.6%) showed ≥ 10% decreases in RFcsa. We did not identify any predictor for significant muscle wasting, however, age was of borderline significance (p = 0.0528). The 28-day mortality rate was higher in patients with significant wasting, but this difference was not statistically significant (30.6% versus 16.7%; p = 0.165). Clinically significant muscle wasting was frequent in our cohort of patients. Patient age was identified as a predictor of borderline significance for muscle wasting. The results could be used to plan future studies on this topic.

Trial registration: ClinicalTrials.gov NCT03865095, date of registration: 06/03/2019.

## Introduction

Acute skeletal muscle wasting and weakness is an important medical problem of critically ill patients^1^. The muscle mass diminishes rapidly during the early phase of a critical illness^2^. Muscle wasting is a result of reduced physical activity, increased protein breakdown, and decreased protein synthesis^3,4^. Loss of muscle mass is associated with poor clinical outcomes. Patients who develop muscle wasting have higher risk of intensive care unit (ICU) acquired weakness^5^, increased length of ICU stay^2^ and loss of muscle mass during the first week of ICU stay is associated with increased 60-day mortality^6^. Weakness and fatigue are persisting symptoms which have a negative impact on the quality of life of critical illness survivors^7,8^.

The identification of predictors for muscle wasting may help to identify the population we have to focus on to improve quality of care and patient-oriented outcomes. However, to the best of our knowledge, only a few published studies were designed to identify predictors for muscle wasting^9,10^.

One of the critical aspects of identifying the predictors for muscle wasting is the correct assessment of muscle mass. This can be challenging in ICU patients because the procedures commonly used for assessing muscle mass, such as manual muscle testing, nerve conduction studies, dual X-ray absorptiometry, computed tomography or magnetic resonance imaging, are rarely useful in critical care settings.ICU patients typically have limited ability to cooperate and follow commands, require trained staff, require invasive procedures, or require patient transport from the ICU. Several studies have documented the use of ultrasonography to estimate muscle quantity and quality at the bedside and have compared these results to measurements of quadriceps muscle thickness in critical care settings^11–13^. Puthucheary et al. proposed the use of the rectus femoris cross-sectional area (RFcsa) as a replacement for measurements of muscle thickness^14^. In contrast to measurements of muscle atrophy based on muscle thickness, changes in RFcsa are directly correlated with changes in muscle strength in critically ill septic patients^15^.

Decrease of RFcsa ≥ 10% is considered as significant and sufficient to affect muscle function^14,16,17^. However, the incidence of significant muscle wasting in critically ill adult patients has been poorly reported to date. We believe that the information about incidence of muscle wasting is importantfor devising optimal patient management strategies in the ICU^14,17^.

Thus, this study aimed to (1) assess the incidence of significant loss of muscle mass in critically ill adult patients; (2) to assess quantitative changes in muscle mass; and (3) to identify any predictors for muscle wasting.

## Results

### Patients

**Figure 1:**
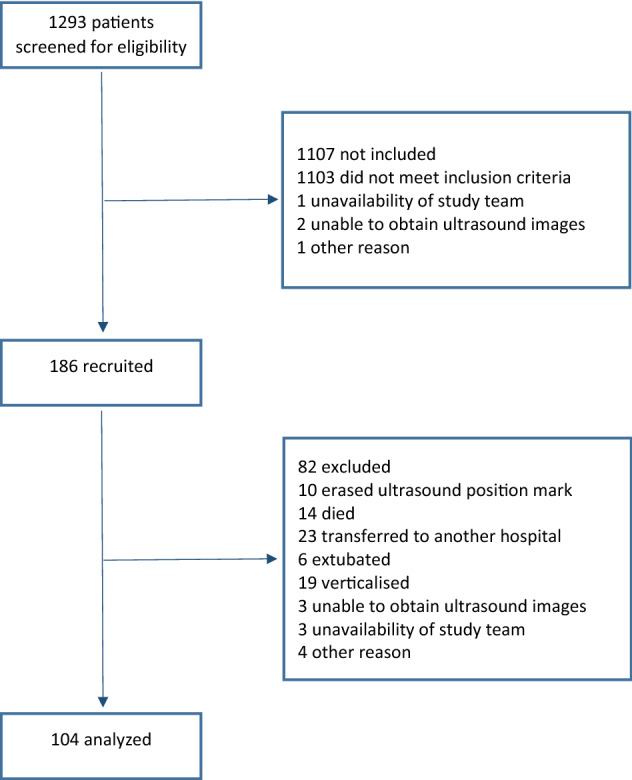
Flow diagram.

A total of 1293 patients was screened during the study period. Of these, 186 patients were initially enrolled based on the inclusion and exclusion criteria. An ultrasound examination was performed to document initial RFcsa within 24 h of ICU admittance. Ultrasound measurements were not performed on day 7 in 82 of these patients due to mortality, transfer to another hospital, and other reasons (Fig. 1), providing us with data from 104 patient cases for analysis. Figure 1 presents the flow diagram for this study. The broad admission categories included medical (n = 41; 39.4%), surgical (n = 3; 2.9%), multiple trauma (n = 44; 42.3%), and neurosurgical (n = 16; 15.4%). Patient characteristics are outlined in Table 1.

**Table 1:** Characteristics of the study population.

Parameter	Statistic	Decrease of RFcsa ≥ 10%		All patients (N = 104)
		Yes (n = 62)	No (n = 42)	
Sex				
Female	n (%)	24 (38.7%)	14 (33.3%)	38 (36.5%)
Male	n (%)	38 (61.3%)	28 (66.7%)	66 (63.5%)
	Fisher's exact test p-value	0.6793		
Age (years)	Mean (SD)	61.0 (17.66)	53.8 (18.75)	58.1 (18.36)
	Wilcoxon p-value	0.0393*		
BMI (kg/m^2^)	Mean (SD)	27.17 (5.587)	27.24 (4.516)	27.20 (5.158)
	Wilcoxon p-value	0.4459		
Reason for hospitalization				
Internal	n (%)	26 (41.9%)	15 (35.7%)	41 (39.4%)
Neurosurgery	n (%)	10 (16.1%)	6 (14.3%)	16 (15.4%)
Polytrauma	n (%)	23 (37.1%)	21 (50.0%)	44 (42.3%)
Surgery	n (%)	3 (4.8%)		3 (2.9%)
	Fisher's exact test p-value	0.4050		
Frailty score				
1	n (%)	6 (9.7%)	3 (7.1%)	9 (8.7%)
2	n (%)	18 (29.0%)	16 (38.1%)	34 (32.7%)
3	n (%)	25 (40.3%)	16 (38.1%)	41 (39.4%)
4	n (%)	10 (16.1%)	5 (11.9%)	15 (14.4%)
5	n (%)	3 (4.8%)		3 (2.9%)
6	n (%)		2 (4.8%)	2 (1.9%)
	Fisher's exact test p-value	0.3767		
APACHE II at ICU admission	Mean (SD)	28.2 (8.36)	26.7 (6.98)	27.6 (7.83)
	p-value of t-test	0.3267		
Enteral nutrition				
Bolus	n (%)	19 (30.6%)	13 (31.0%)	32 (30.8%)
Continuous	n (%)	43 (69.4%)	29 (69.0%)	72 (69.2%)
SOFA at ICU admission	Mean (SD)	10.0 (2.90)	9.1 (2.88)	9.6 (2.91)
	Wilcoxon p-value	0.2066		

*APACHE II* Acute Physiology And Chronic Health Evaluation II, *BMI* body mass index, *ICU* intensive care unit, *SOFA* sequential organ failure assessment.

### Measurement of rectus femoris cross-sectional area

The mean RFcsa (95% CI) on day 1 was 2.638 (2.286–2.990) cm^2^ for the group of patients with RFcsa measurements that decreased by ≥ 10% and 2.333 (1.967–2.699) for the group that exhibited a < 10% decrease in RFcsa. The mean (95% CI) RFcsa on day 7 was 1.986 (1.683–2.289) cm^2^ versus 2.355 (2.001–2.710) cm^2^ in the groups exhibiting decreases of ≥ 10% and < 10%, respectively. These results are shown in Table 2.

**Table 2:** Measurement of rectus femoris cross-sectional area (RFcsa).

Parameter	Statistic	Decrease in RFcsa ≥ 10%	
		Yes (n = 62)	No (n = 42)
RFcsa on Day 1 (cm^2^)	Mean (SD)	2.638 (1.3852)	2.333 (1.1746)
	95% CI	2.286–2.990	1.967–2.699
RFcsa on Day 7 (cm^2^)	Mean (SD)	1.986 (1.1929)	2.355 (1.1381)
	95% CI	1.683–2.289	2.001–2.710

### Outcomes

We evaluated 104 patients in this study. Sixty-two of these patients (59.6%) exhibited a ≥ 10% decrease in RFcsa between day 1 and day 7 while in the ICU. The mean (95% CI) SOFA score on day 7 was 6.9 (5.8–7.9) in patients who exhibited a < 10% decrease in RFcsa and 7.6 (6.6–8.6) in patients who exhibited ≥ 10% decreases in RFcsa. The mean duration of mechanical ventilation was 12.3 (9.9–14.7) and 11.4 (9.4–13.4) days for patients with decreases in RFcsa of < 10% and ≥ 10%, respectively. The mean length of stay in the ICU was 18.8 (16.6–21.1) and 17.0 (15.1–18.9) days for these two groups, respectively. The mean 28-day mortality was 16.7% in patients that exhibited a < 10% decrease in RFcsa and 30.6% in patients that exhibited a ≥ 10% decrease in RFcsa. Although mortality nearly doubled in the group of patients that exhibited a ≥ 10% decrease in RFcsa compared with those that exhibited a < 10% decrease in RFcsa. None of these differences achieved statistical significance (see p-values in Table 3). The outcome data are summarized in Table 3.

**Table 3:** Outcome characteristics of patients.

Parameter	Statistic	Decrease in RFcsa ≥ 10%		p-value*
		Yes (n = 62)	No (n = 42)	
Difference in SOFA score between Day 1 and Day 7	Mean (SD)	−2.4 (3.64)	−2.2 (4.06)	0.6258
	95% CI	−3.3 to −1.5	−3.5 to −0.9	
Mechanical ventilation duration (days)	Mean (SD)	11.4 (7.87)	12.3 (7.73)	0.3457
	95% CI	9.4–13.4	9.9–14.7	
ICU length of stay (days)	Mean (SD)	17.0 (7.51)	18.8 (7.20)	0.1675
	95% CI	15.1–18.9	16.6–21.1	
28-day mortality				
Alive	n (%)	43 (69.4%)	35 (83.3%)	0.1654
Dead	n (%)	19 (30.6%)	7 (16.7%)	

*SOFA* sequential organ failure assessment.

*p-value of Wilcoxon–Mann–Whitney U-test for continuous parameters and Fisher’s exact test for mortality.

### Predictors for a ≥ 10% decrease of RFcsa

All characteristics listed in Table 1 were explored as potential predictors for their relationship with RFcsa reduction graphically and by means of univariate logistic regression. Further forward stepwise selection in a multivariate logistic regression model was used to identify significant predictors. Patient age was the only parameter identified as a significant predictor, however on borderline statistical signifikance, for a ≥ 10% decrease in RFcsa. The results of this logistic regression analysis are shown in Table 4. The odds ratio suggests that, for each one-year increase in age, the odds that an ICU patient will experience a ≥ 10% decrease in RFcsa increase by 2%.

**Table 4:** Significant predictors for decreases in RFcsa ≥ 10%.

Parameter	Statistic	Value
Age (years)	Odds ratio	1.022
	95% Wald CI	1.000–1.044
	p-value (Wald)	0.0528

## Discussion

Muscle wasting contributes significantly to weakness among patients who have recovered from a critical illness and can have a negative impact on a patient’s quality of life^7^. Recent research has focused on identifying adequate techniques of assessment of muscle wasting as well as factors contributing to muscle wasting in critical care settings. Ultrasound measurements of muscle mass and other parameters of different muscles were investigated and reported in critically ill patients recently^18^. Ultrasound is thus a well-established method for achieving this goal. For example, Mourtzakis et al.^19^ reported the outcomes of 11 observational and three interventional studies that used ultrasound measurements to assess muscle wasting in critically ill patients. Ultrasound has specifically been used to assess muscle mass by measuring the RFcsa, which could replace methods that measure muscle thickness^14^. Ultrasound measurements of RFcsa are feasible at the bedside, require no patient cooperation, and show good intra- and interobserver agreement^20,21^.

Previous studies that assessed muscle wasting using RFcsa concluded that a > 9.24% decrease in RFcsa was significant^16,17^. Observational and interventional studies aimed at the preservation of muscle mass have used a cut-off of a ≥ 10% reduction in RFcsa^14,22^. To our knowledge, no studies in critical care settings that have used a muscle-wasting criterion of a > 10% reduction in RFcsa as a cut-off for significant muscle wasting have reported its incidence. In this study, the incidence of significant muscle mass wasting was 59.6%, which is similar to the incidence reported in cardiac surgery^16,17^.

While loss of muscle mass has frequently been observed in association with critical illness, the relationship of muscle wasting degree to clinical outcomes remains unclear.

Only a few studies reported the effect of loss of muscle mass on clinical outcomes and none of these studies focused on clinical outcome measures as primary endpoints^16,22^. Bloch et al. conducted an observational study in high-risk cardiovascular surgery patients and found no statistical differences in the length of ICU or hospital stay between patients with or without muscle wasting, which was defined as reduction of RFcsa > 9.24%^16,18^. We obtained similar results in this study. The relationship between significant muscle wasting and mortality in adult patients in critical care settings also remains unknown. Our results revealed a nearly two-fold increase in 28-day mortality in patients with significant muscle wasting (30.6% versus 16.7% in the groups with ≥ 10% and < 10% decreases in RFcsa, respectively). Although this difference was not statistically significant, the study sample size was not pre-set to evaluate this question.

Patients frequently lose muscle mass in critical care settings; for example, one study showed that patients with multiple organ failure experienced a 15.7% reduction in RFcsa during their first 7 days in the ICU^2^. In study by Mayer et al. found decrease of 18.9% of the RFcsa^23^. In our study, the mean decrease in RFcsa over 7 days in all enrolled ICU patients was 14.9%. The higher reduction of RFcsa in study by Meyer may be related do differnet study population, as the majority of patients in that study were admitted to medical ICU.

Predictors for muscle wasting remain poorly understood. Puthucheary et al.^2^ found that age, bicarbonate level at hospital admission, and the ratio of PaO_2_ to FiO_2_ were associated with a > 10% decrease in RFcsa measured on day 10 of an ICU stay. Mayer et al.^23^ recently reported age among risk factors of muscle sit-to-stand performance at hospital discharge in patients with critical illness. In our study, age was found of borderline signifikance as a predictor for a > 10% decrease in RFcsa measured on day 7 of an ICU stay. Larger study is needed to prove this finding.

There were several limitations associated with this study. First, the small sample size precludes a full evaluation of the relationship between the loss of muscle mass and 28-day mortality. Second, the patient cohort surveyed in this study is highly heterogeneous. For example, we enrolled significantly more male than female patients, and our results may therefore not be valid for both sexes. The reasons patients were admitted to the ICU were also not evenly represented, as most patients were admitted for internal complications or trauma, whereas few were admitted for surgical complications. Third, the site of ultrasound measurement did not follow any generally accepted protocol as none such exists. On the other hand, as shown in a review by Nascimento^18^, the most frequently used site for assessing quadriceps muscle is 2/3 of the anterior iliac spine—patella distance. However, we found in the preparation phase of the study that with this protocol we were not able to obtain a cross-section of rectus femoris muscle in total. This was due to the type of ultrasound probe available. Fourth, the side where the RFcsa measurement was performed was not standardized across all patients and was instead subjectively chosen by the investigator. Fifth, we did not document the hand dominance of each patient in this cohort. We therefore may have measured RFcsa on the patient’s non-dominant side, leading to potential overestimation of muscle wasting. We finally recognize the potential for selection bias, as primary patient selection was based on the investigator’s subjective assessment of which individuals were likely to need mechanical ventilation for more than 48 h after ICU admission.

Our results suggest that many adult patients in critical care settings experience a significant loss of muscle mass, defined as a ≥ 10% decrease in RFcsa over seven days of ICU care. Our results could be used to determine the required sample sizes for randomized controlled trials that further examine muscle wasting in critical care patients and identify strategies for mitigating muscle loss.

The generalizability of this study is somewhat limited due to the observational design and the fact that it was carried out at a single medical center.

Significant loss of muscle mass was assessed by ultrasound measurements of RFcsa in 104 critically ill adult patients. Patient age at admission was associated with a risk of developing clinically significant muscle wasting. Mortality rates were two-fold higher in patients with significant muscle wasting (≥ 10% over the first seven days in the ICU) than among those without significant wasting, though this difference in mortality was not statistically significant.

Muscle wasting occurs rapidly^2^ and has an important role in development of ICU acquired weakness^7^. It also has a negative impact on clinical outcomes as functional disability at ICU discharge^9^. Earlier and greater loss of muscle mass is associated with prolonged mechanical ventilation, ICU acquired weakness and in-hospital mortality^13^. Our result showed that significant muscle wasting is frequent among critically ill patients. The results of this study could be used to plan future studies evaluating strategies to prevent muscle wasting and thus positively affect the outcomes of critically ill patients, e.g. nutrition regimens or physiotherapy.

## Materials and methods

### Study design, setting, and participants

This single-center prospective observational cohort study is registered at ClinicalTrials.gov (NCT03865095). Ethical approval for this study (Ethical Committee No. 05-130219/EK) was provided by the Ethical Committee of University Hospital Brno, Brno, Czech Republic (Chairperson PharmDr.S.Kozakova, MBA) on 13 February 2019. This work was performed in accordance with relevant guidelines and regulations and in accordance with the Declaration of Helsinki. Inform consent was obtained from all enrolled patients. If a patient has impaired consent capacity, the informed consent was obtained from a legal representative. If a legal representative was not established or known at the time of enrolment, the informed consent was obtained from a physician who was independent on study conduct and familiar with study protocol. All patients admitted to one of the four ICUs of the Department of Anesthesiology and Intensive Care Medicine of University Hospital Brno from March 2019 to September 2020 were screened for eligibility for enrolment in this study. Inclusion criteria at ICU admission included ≥ 18 years of age and the physician’s subjective evaluation that the patient would require mechanical ventilation for at least 48 h. Exclusion criteria included age < 18 years, a Clinical Frailty Score > 7 prior to admission, a past medical history of neuromuscular disease, amputated lower extremities, prior trauma to the lower extremities involving thighs and inability to cooperate with ultrasound examinations. Patients whose ultrasound measurement was not feasible on day 7 were excluded from the final analyses, reasons for exclusion were provided (Fig. 1).

### Variables, data sources, and measurements

The primary outcome of the study was the quantitative assessment of decreased RFcsa, defined as the percentage reduction in RFcsa based on the results of ultrasound evaluation on day 1 and day 7 of the patient’s ICU stay. We considered a decrease in RFcsa ≥ 10% to be clinically significant muscle wasting. The secondary outcome of the study was 28-day mortality. We also examined predictors for a clinically significant decrease in RFcsa.

Ultrasound measurements (Vivid S6, GE Healthcare) of RFcsa were performed by the same investigator within the first 24 h after ICU admission (day 1) and again on day 7 of the patient’s ICU stay. The side of measurement was left at the discretion of the ultrasonographer. The physical point at which the ultrasound measurement was performed was drawn on the patient’s skin as a line perpendicular to the long axis of the thigh, three-quarters of the distance between the anterior superior iliac spine and the middle of the upper part of the patella. A linear ultrasound probe with a frequency of 3–9 MHz was placed on the marked line perpendicular to the skin using an excess of gel and minimal pressure. Six images of the rectus femoris muscle cross were collected and stored. An ultrasound machine tool for area measurement was used for RFcsa measurements. The inner side of the fascia of the rectus femoris muscle was manually traced in each image. For the calculation of average value of ultrasound measurements we adopted the method described by Turton et al.^24^, we omitted the smallest and largest values and calculated the average of the remaining four values. The member of the study team who performed the examination entered the ultrasound measurements into the case report form. To assess the reproducibility of our ultrasound measurements we performed separate analysis of intra- and interobserver agreement in 25 consecutive patients. The measurements were performed by two observers. Observer A repeated the measurement 30 min after the first one, and observer B performed the measurement independently of observer A and was blinded to the results of observer A. The inter-class correlation coefficients for intra- and interobserver agreement showed excellent correlation^21^.

All study-related patient data (age, sex, APACHE II score, SOFA score, Frailty scale value, and ICU Mobility score on admission and day 7) were obtained from medical records and entered into the case report form by a study team member.

### Bias and sample size

We cannot rule out selection bias in this study, as one of the primary inclusion criteria (i.e., the physician’s personal assessment of the need for 48 h of mechanical ventilation) was subjective in nature. The likelihood that a patient would need mechanical ventilation for > 48 h was assessed after discussion between the investigator and the senior ICU physician. This discussion took into account findings from initial diagnostic work-up and the reaction to therapeutic interventions. Because we were unable to locate any previous studies that documented the incidence of significant muscle wasting in critically ill adult patients, we were unable to use previous data to determine the relevant sample size. However, we did note that Bloch et al.^17^ reported significant muscle wasting (defined as a decrease in RFcsa of more than 10% at day 7 of an ICU stay) in 55% of patients who had undergone cardiothoracic surgery. Based on these findings, we estimated that 100 patients would provide the appropriate sample size for determining the incidence of significant muscle wasting. Given the possibility that many of the enrolled patients might not fully complete the study protocol, we increased the number of participants to 175. To cover potential loss of follow-up, the number of participants was increased to 186.

### Statistical analysis

Continuous demographic data and baseline patient characteristics are presented as means with standard deviations (SDs). Continuous outcome characteristics and measurements of RFcsa are presented as means and 95% confidence intervals (CIs) with SDs. Categorical characteristics are summarized using absolute counts and percentages. There were no missing RFcsa measurements, outcome characteristics, demographic, or baseline data for any of the 104 patients we evaluated.

All potential predictors were examined using both descriptive statistics and forward stepwise selection in a logistic regression model. Predictors were retained in the model if they had p-value < 0.1. A logistic regression model that included change in RFcsa as a dependent binary variable and predictors that were retained after forward selection as predictors were constructed to estimate odds ratios and 95% Wald CIs.

The relationships between patient characteristics and decreases in RFcsa were examined using Wilcoxon–Mann–Whitney U-tests. The relationship between a decrease in RFcsa and 28-day mortality was evaluated using Fisher’s exact test with an alpha level of 0.05. All statistical analyses were conducted using SAS 9.4 (SAS Institute, Cary NC).
